# Patient-reported outcomes of laparoscopic magnetic sphincter augmentation for gastro-oesophageal reflux disease

**DOI:** 10.1308/rcsann.2023.0051

**Published:** 2023-08-23

**Authors:** D Nehra, CDM Clements, SL Bezzaa, Y Tabbakh, CM Walsh

**Affiliations:** ^1^ Epsom and St Helier University Hospitals NHS Trust, UK; ^2^ Imperial College London, UK

**Keywords:** Gastroenterology, Digestive system surgical procedures, Fundoplication, Gastroesophageal reflux, Oesophageal sphincter, lower

## Abstract

**Introduction:**

Gastro-oesophageal reflux disease (GORD) is a chronic progressive disease, associated with substantial clinical and economic burden. Proton pump inhibitors (PPIs) are considered first-line treatment; however, there are concerns around the long-term impact of their usage. Surgical treatment with Nissen fundoplication can be considered but, because of the potential side effects, few patients undergo surgery and there remains a substantial therapeutic gap within the current treatment pathway. Laparoscopic magnetic sphincter augmentation (MSA) using the LINX^®^ device is an alternative surgical approach.

**Methods:**

The objective of this study was to investigate patient-reported outcomes following laparoscopic MSA surgery using the LINX^®^ device in a UK setting. A retrospective questionnaire obtained data regarding postoperative symptoms, medication use and patient satisfaction.

**Results:**

Out of 131 patients surveyed, 97 responses were received, with a minimum follow-up time of 1 year. In those who reported heartburn and regurgitation preoperatively, improvement was reported in 93% (84/90) and 90% (86/96) of patients, respectively. Eighty-eight per cent (73/83) of patients were able to completely stop or reduce their medication by at least 75%. Seventy-seven per cent (73/95) of patients were “very satisfied” or “satisfied”.

**Conclusions:**

This study is the first to present patient-reported outcomes of MSA using the LINX^®^ device for patients with GORD in the UK. It demonstrates that the device has favourable outcomes and could effectively bridge the current therapeutic gap that exists between PPI medication and Nissen fundoplication.

## Introduction

Gastro-oesophageal reflux disease (GORD) is a common, chronic progressive disease with substantial clinical and economic burden.^1^ GORD causes a variety of symptoms typical of reflux but has also been associated with longer-term complications including oesophagitis and stricture development, in addition to intestinal metaplasia (Barrett’s oesophagus) and oesophageal adenocarcinoma.^2–5^ The recommended first-line treatment for medical management of symptoms of GORD is protein pump inhibitors (PPIs).^6^ PPIs are the second most prescribed drug in the UK with associated costs of around £300 million per year.^7^ Although these can alleviate heartburn symptoms, PPIs do not directly treat the dysfunction of the lower oesophageal sphincter.^8,9^ In our experience, many patients are concerned with the impact of long-term PPI use, including potential side effects such as osteoporosis and renal impairment.^10,11^

In patients who have refractory symptoms, develop complications despite medication or are intolerant to medication, surgical treatment with fundoplication can be considered.^6^ There are two key types of laparoscopic fundoplication: Nissen (total) and partial fundoplication.^12^ In the authors’ experience, Nissen fundoplication is considered to be the gold-standard approach. However, this procedure has been associated with side effects such as bloating, dysphagia and an inability to belch or vomit.^13^ In addition, there may be concerns regarding durability and the risk of requiring repeat surgical procedures.^14^ Therefore, many patients do not undergo treatment with Nissen fundoplication, leaving a substantial unmet need for treatment of patients with refractory symptoms of GORD.

Laparoscopic magnetic sphincter augmentation (MSA) using the LINX^®^ device is an alternative surgical approach to fundoplication for the treatment of GORD.^15^ Several studies have shown that MSA has similar symptom resolution, improved quality of life, reduced operative time, as well as fewer patient complications and side effects compared with Nissen fundoplication.^16,17^ With MSA, no alteration to stomach anatomy is required, unlike with fundoplication, and patients retain the ability to belch and vomit.^17,18^ MSA also uses a standardised approach, which is anticipated to result in consistent outcomes.^19,20^ The LINX^®^ device is implanted laparoscopically and patients are usually discharged the same day, or one day postoperatively.^19^ A 2023 National Institute for Health and Care Excellence (NICE) guidance document reported that the evidence for the safety and efficacy of LINX^®^ is adequate to support the MSA procedure.^21^

### Study objective

The objective of this study was to investigate the patient-reported outcomes following laparoscopic MSA surgery using the LINX^®^ device in a UK setting. This is the first study to report patient outcomes with a minimum of 1-year follow-up in the UK.

## Methods

### Study design

This was a retrospective study of all patients who underwent MSA surgery with a LINX^®^ device between 2012 and 2021 at a single institution in the UK (Epsom and St Helier University Hospitals NHS Trust).

GORD was diagnosed using a standard clinical approach including assessment of symptoms, endoscopy, manometry and/or pH studies as deemed clinically appropriate. MSA surgery with the LINX^®^ device was offered to all patients considered suitable for anti-reflux surgery. Alternatively, patients were offered partial fundoplication. Patients with a hiatal hernia >6cm in diameter were not considered to be suitable for MSA surgery with the LINX^®^ device.

Eligible patients for this study were those who had undergone MSA surgery with the LINX^®^ device. Starting at study commencement in September 2019 until data cut-off in June 2022, questionnaires were sent to all eligible patients once they reached 1-year post-MSA surgery. Non-responders to the questionnaire were followed-up via a telephone call.

The study was designed to align with the Strengthening the Reporting of Observational Studies in Epidemiology (STROBE) guidelines, using the combined checklist. This provides guidance on reporting observational studies, to improve the quality, value and safety of healthcare.^22^

### Study outcomes

The questionnaire captured data for three key areas: presence of symptoms and side effects, medication use and patient satisfaction. The full patient questionnaire, including all the outcomes investigated, is included in the supplementary data available online.

#### Symptoms and side effects

Symptoms of heartburn, regurgitation, sore throat, hoarseness or chronic cough due to reflux were investigated by asking patients whether their symptoms had improved after surgery (yes/no). If symptoms had improved, the level of improvement could then be reported as 0%–25%, 25%–50%, 50%–75% or >75%. Similarly, symptoms of dysphagia were assessed by asking whether patients experienced dysphagia after the procedure (yes/no) and, if so, for how long in months. The severity of dysphagia was assessed by the response options of mild (one or two episodes a week), moderate (more than two episodes per week) or severe (most meals). The presence of side effects, including bloating or gassy feelings, ability to belch and ability to vomit were captured as yes or no.

#### Medication use

Medication use was assessed in terms of both discontinuation and reduction. Patients were able to report whether they were still taking their medication for reflux and, if so, whether they were taking the same amount as before the surgery, or if they had reduced their use by 25%, 50% or 75%.

#### Patient satisfaction

Patient satisfaction was assessed using a five-point scale (very satisfied, satisfied, neutral, unsatisfied, very unsatisfied) and asking whether the patient would recommend the surgery to others (yes/no). Patients were also given the opportunity to provide comments within a free-text box, and these responses were categorised manually and independently by three of the authors. Each free-text response was labelled overall positive, neutral or overall negative, on a three-point scale.

#### Safety

Safety data were collected using clinical notes for all 131 patients who underwent the MSA surgery during the study period, regardless of response to the questionnaire.

#### Missing data

Where data were missing for an outcome, the patient was excluded from analysis for that specific outcome. The total patient population considered for analysis for each outcome therefore varies and is reported accordingly.

## Results

### Patient population

The questionnaire was sent to 131 patients in total and 97 responses were received, representing a 74% response rate.

The time between surgery and being sent the questionnaire varied from a minimum of 1 year, up to 6 years 10 months for the first patient in the study who underwent MSA surgery. The age range of patients was 18–71 years (median: 49 years; *n* = 90), with a male to female ratio of 0.96:1 (*n* = 90); patient demographics are presented in Table 1.

**Table 1:** Patient demographics

Demographic	Patients (*n* = 90*)
Male, % (*n*)	49 (44)
Female, % (*n*)	51 (46)
Age (years), median	49
Age (years), range	18–71

*Demographic data were missing for seven patients because questionnaire responses could not be linked with these patients owing to missing information

Thirty per cent (27/90) of patients were discharged on the same day as undergoing the procedure. Most patients were discharged 1 day post operation (61%, 55/90). Eight patients were discharged 2 or more days post operation. Discharge data were missing for seven patients.

LINX^®^ device size ranged from size 13 to size 16 (*n* = 90). The size of the LINX^®^ device used is shown in Table 2.

**Table 2:** Size of LINX^®^ device used

LINX^®^ device	Patients (*n* = 90*)
Size 13, % (*n*)	2 (2)
Size 14, % (*n*)	20 (18)
Size 15, % (*n*)	41 (37)
Size 16, % (*n*)	37 (33)

*Data were missing for seven patients

The presence of a preoperative hiatal hernia was reported for each patient. In the cases in which a hiatal hernia was present, the size was also reported, as presented in Table 3. Intraoperative hernia repair was conducted in 91% of patients (81/89).

**Table 3:** Presence and size of hiatal hernia

Presence and size of hernia*	Patients (*n* = 88^†^)
No hiatal hernia, % (*n*)	18 (16)
Small (<2cm), % (*n*)	41 (36)
Medium (2–3cm), % (*n*)	30 (26)
Large (>3), % (*n*)	11 (10)

*Size of hiatal hernia was based on clinical notes. Where a size in centimetres was reported, the authors categorised the hernia size as follows: <2cm, small; 2–3cm, medium; >3cm, large

^†^Data were missing for nine patients (including one patient with hiatal hernia of unknown size who underwent hernia repair).

### Symptoms and side effects

**Figure 1:**
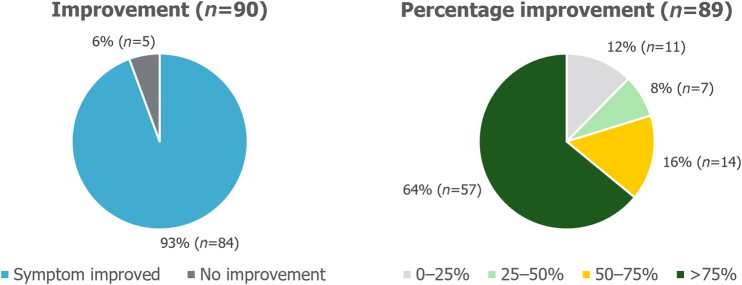
Postoperative improvement in heartburn symptoms. Non-responders were excluded from the analysis.

**Figure 2:**
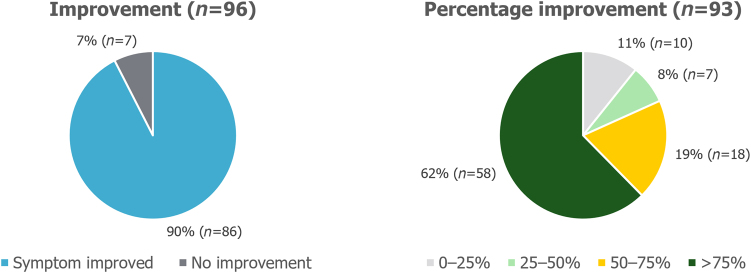
Postoperative improvement in regurgitation symptoms. Non-responders were excluded from the analysis.

**Figure 3:**
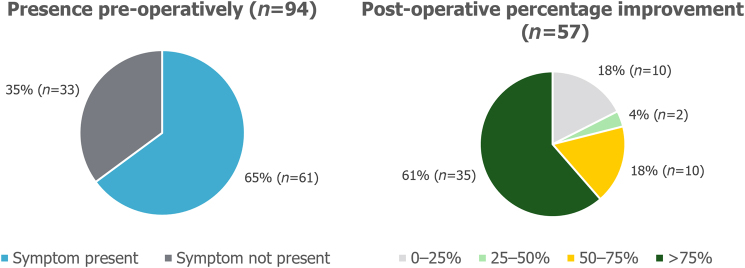
Postoperative improvement in sore throat symptoms. Non-responders were excluded from the analysis.

Out of the patients who reported that the symptom was present preoperatively, 93% (84/90) of patients with heartburn and 90% (86/96) of patients with regurgitation reported that their symptom had improved after surgery. These symptoms had improved by >75% compared with before the surgery in 64% (57/89) and 62% (58/93) of patients for heartburn and regurgitation, respectively, as presented in Figures 1 and 2. Before the surgery, 65% (61/94) of patients experienced symptoms of sore throat, hoarseness or chronic cough. Postoperatively, 61% (35/57) of patients reported that their symptoms had improved by >75% (Figure 3).

Patient-reported postoperative side effects are described in Table 4. After the surgery, 91% (87/96) of patients were able to belch and, of the patients who needed to vomit, 84% (61/73) reported that they were able to do so.

**Table 4:** Patient-reported postoperative side effects

Outcome	Yes	No
Presence of postoperative side effects, % (*n*)		
Bloating, *n* = 96*	53 (51)	47 (45)
Dysphagia, *n* = 95*	93 (88)	7 (7)
Postoperative belching and vomiting capability, % (*n*)		
Ability to belch, *n* = 96*	91 (87)	9 (9)
Ability to vomit, *n* = 73*	84 (61)	16 (12)

*Non-responders were excluded from the analysis

**Figure 4:**
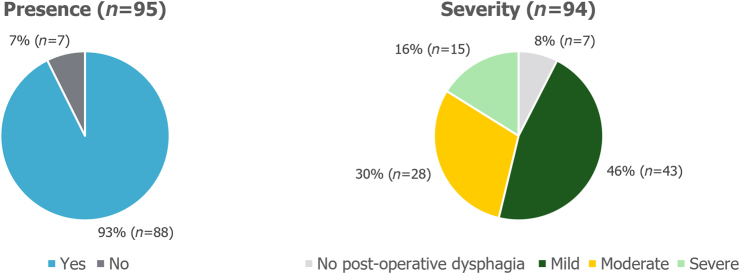
Presence and severity of postoperative dysphagia. Non-responders were excluded from the analysis.

Dysphagia was experienced in 93% (88/95) of patients postoperatively, but in 46% (43/94) of patients this was mild in severity. In 30% (28/94) of patients, postoperative dysphagia was moderate in severity and in 16% (15/94) of patients it was reported to be severe (Figure 4).

**Figure 5:**
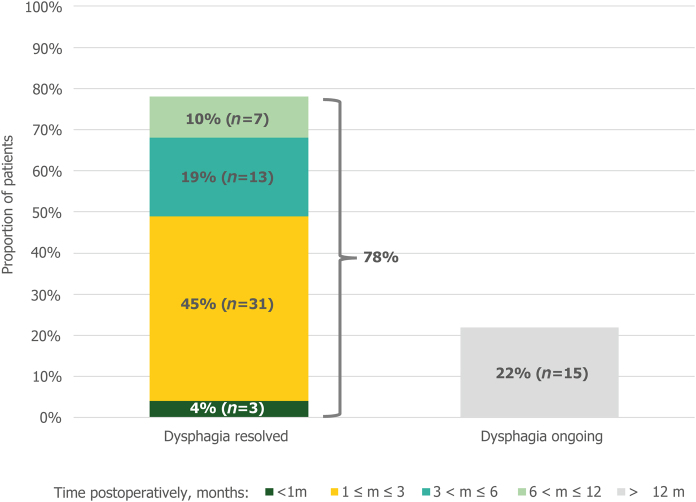
Duration of postoperative dysphagia (*n* = 69). Non-responders and patients with no postoperative dysphagia were excluded from the analysis.

Sixty-nine patients reported the duration of their postoperative dysphagia. In 49% (34/69) of patients, this postoperative dysphagia was transient and resolved within 3 months, as demonstrated in Figure 5. At the time of survey, 22% (15/69) patients were still experiencing postoperative dysphagia.

### Medication use for reflux

**Figure 6:**
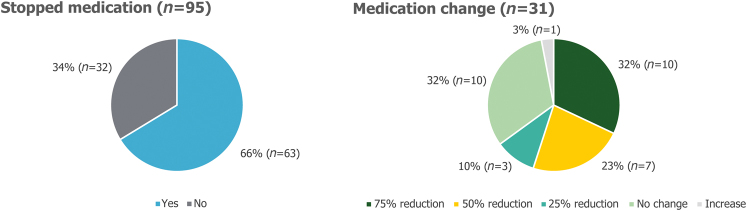
Postoperative change in reflux medication. Non-responders were excluded from the analysis.

Sixty-six per cent (63/95) of patients were able to completely stop taking medication for reflux. Twelve per cent (10/83) of patients were able to reduce their medication intake by 75%; 8% (7/83) were able to reduce their intake by 50%; and 4% (3/83) were able to reduce their intake by 25%, as presented in Figure 6.

### Patient satisfaction

**Figure 7:**
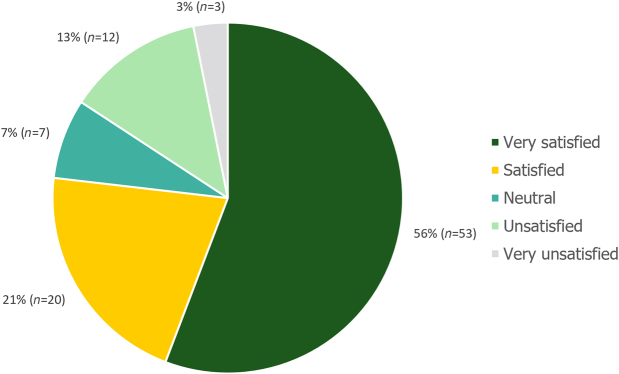
Patient satisfaction (*n* = 95). Non-responders were excluded from the analysis.

When asked to rate how satisfied they were with their present condition, 77% (73/95) of patients were either “very satisfied” or “satisfied”, and 3% (3/95) were “very unsatisfied”. These patient satisfaction results are presented in Figure 7.

Of the fifteen patients who reported that they were “unsatisfied” or “very unsatisfied” with their present condition, six patients reported ineffectiveness as the primary reason for their dissatisfaction, three patients reported dysphagia and three reported recurrence. Lack of preoperative information and slow recovery time were reported by one patient each, and no further details were provided for one patient.

When asked if they would recommend the surgery to others, 90% (84/93) of patients said they would.

Free-text comments were provided by 65% (63/97) of patients. Of these comments, in 62% (39/63) of patients the response was categorised as overall positive. In 24% (15/63) of patients the responses were categorised as neutral, and in 14% (9/63) of cases the responses were categorised as overall negative.

### Safety

Out of the 131 patients who underwent MSA surgery during the study period, including the non-responders to the survey, adverse events were reported in nine cases. This included two cases of intraoperative pneumothoraces, one postoperative bleed and one readmission with an intra-abdominal collection. Delayed complications included one case where a food bolus lodged in the distal oesophagus and one case with a suspected vagal nerve injury causing gastric stasis. Persistent dysphagia lasting longer than 1 year occurred in three patients who underwent endoscopic dilatation; however, in two cases dysphagia remained unresolved and the LINX^®^ device was removed. A further explant of the device was performed for a patient who requested removal for side effects, resulting in a total explant rate of 2.3% (3/131).

## Discussion

This study is the first to present patient-reported outcomes of MSA using the LINX^®^ device for patients with GORD in the UK. It demonstrates that the device has favourable patient-reported outcomes and is effective at reducing the symptoms of GORD.

The first-line treatment of GORD, for medical management of symptoms, is PPIs.^6^ However, around 40% of patients who are taking PPI medication have ongoing GORD symptoms.^16^ Laparoscopic fundoplication is a surgical treatment option for these patients, but Nissen fundoplication can be associated with side effects, such as bloating, dysphagia and an inability to belch or vomit.^13^ Because of the risks and potential complications associated with the procedure, patient symptoms must be considered severe enough to warrant treatment using fundoplication. Therefore, in clinical practice, fewer than 1% of patients with a diagnosis of GORD undergo Nissen fundoplication surgery to treat their symptoms, resulting in a substantial proportion of patients who are burdened with ongoing symptoms.^23^ Although partial fundoplication is an alternative surgical option for patients, there remains a substantial therapeutic gap within the current treatment pathway. The patient-reported outcomes presented in this study suggest that the LINX^®^ device could be used to bridge this gap.

The results show that the device provides a high resolution rate of GORD symptoms, with over 90% of patients reporting an improvement in regurgitation and heartburn symptoms. It has been previously reported that MSA with the LINX^®^ device had similar improvements in quality of life and symptom relief compared with Nissen fundoplication, and was found to be more effective at controlling symptoms than PPIs.^13,16,17,24,25^

Previous literature also suggests that MSA with the LINX^®^ device is associated with fewer side effects than Nissen fundoplication, where often patients are not able to belch or vomit after the procedure.^13,24^ In this study, the majority of patients retained the ability to belch (91%, 87/96) and, of patients who reported the need to vomit, 84% (61/73) were able to do so.

This study demonstrates that treatment using the LINX^®^ device resulted in an acceptable rate of side effects. The early postoperative dysphagia rates, although high, are as expected following MSA and resolved quickly in most cases within a year of surgery. Dysphagia was mild in 46% of cases (43/94). Evidence from the literature suggests that the rate of dysphagia is similar for Nissen fundoplication.^13,24^

The impact of the surgical intervention on medication use was also demonstrated in this study. More than half of all patients (88%, 73/83) were able to completely stop medication for reflux, or reduce their medication use by at least 75%. This is likely to be beneficial for patients’ overall health, because long-term PPI use can be associated with many possible side effects.^9,26–28^

In this study, the device was well tolerated; adverse events were reported in nine cases and there were only three cases where the device was removed. There were no reported migrations or erosions. These findings provide additional evidence for the safety of the LINX^®^ device.

The results presented here, from a UK population, align with multiple studies from the USA that present the feasibility and effectiveness of MSA with the LINX^®^ device.^18,20,29–32^ It has been shown that, up to 5-years post procedure, MSA is effective, there are minimal long-term complications, and it has an acceptable safety profile.^33–36^

### Study limitations

This retrospective, patient-reported outcome study has some limitations to note. As a result of the variable follow-up time between the procedure and the questionnaire being completed, there is inconsistency in the length of follow-up across all patients. In some cases, it is anticipated that a longer follow-up may be required to see the full benefit of the procedure. In addition, not all patients who responded to the survey answered every question, and so there are missing data for some of the outcomes. Given the length of the study, which covered procedures undertaken over a 10-year period, the techniques and perioperative management evolved over time. In our experience, by the end of the study period, the device used was one or two beads larger than the average size of device used in earlier procedures, larger hiatal hernias were considered for the procedure, and there was a move towards same-day discharge. Despite these changes in surgical management, patient satisfaction following the procedure remained high.

A novel questionnaire was utilised, which may limit comparison with other studies. However, the questionnaire was designed to be concise and clear for ease of completion by patients at home, with the aim of optimising response rates.

Although this study considered patient-reported improvement in symptoms, and a qualitative reduction in medication, no clinical outcomes were recorded apart from those collected as part of standard practice. To further validate the findings of this study, future research could focus on collecting additional clinical outcomes during the follow-up period. Although the data were collected from a single centre, the minimally invasive surgical procedure for the placement of the LINX^®^ device is standardised, and so it is anticipated that the results of this study would be reflected at other centres.

We note that patient-reported outcome measures for anti-reflux surgery (including MSA with the LINX^®^ device) are currently being collected in the prospective National Hiatal Surgical Registry, of which the authors are major contributors. This database is anticipated to provide further patient-centric outcomes for anti-reflux surgical approaches.^37^

One of the key strengths of this study is that meaningful patient-reported outcomes were collected. In this way, the results show the direct impact MSA surgery with the LINX^®^ device had on patient symptoms, side effects, medication and overall satisfaction. The study was conducted in the UK and captured outcomes after a longer period than previous studies.

## Conclusions

In conclusion, this study showed a broad positive impact of MSA with the LINX^®^ device on patient-reported outcomes. When analysing the data covering a period of 10 years, the majority of patients reported improvements in regurgitation and heartburn symptoms and were able to discontinue or reduce their reflux medication. The procedure also had high patient satisfaction rates, with 90% of patients reporting that they would recommend LINX^®^. These results add a patient-centric perspective to the current evidence base, indicating that the procedure could be an effective way to bridge the current therapeutic gap that exists between PPI medication and Nissen fundoplication. The LINX^®^ device could provide an alternative treatment option for patients whose symptoms are not well controlled with medication, or for whom long-term PPI medication is not an option.

## Data sharing statement

The data that support the findings of this study are available from the corresponding author upon reasonable request.

## Conflicts of interest

Mr D Nehra is a preceptor for magnetic sphincter augmentation (MSA) procedures on behalf of Johnson & Johnson. The other authors do not have any disclosures.

## Funding

Support for third-party writing assistance for this article, provided by Rebecca Yusaf, MA VetMB MRCVS, and Eleanor Mackle, PhD, from Costello Medical, UK, was funded by Johnson & Johnson in accordance with Good Publication Practice (GPP3) guidelines (http://www.ismpp.org/gpp3). Johnson & Johnson were not involved in the conduct of the study, interpretation or analysis of data, or overall messaging.

## Author contributions

DN, CDMC, SLB, YT, CMW: substantial contributions to study conception and design. DN, CDMC, SLB, YT, CMW: substantial contributions to analysis and interpretation of the data. DN, CDMC, SLB, YT, CMW: drafting the article or revising it critically for important intellectual content. DN, CDMC, SLB, YT, CMW: final approval of the version of the article to be published.
